# *Escherichia coli* O104:H4 Infections and International Travel

**DOI:** 10.3201/eid1803.111281

**Published:** 2012-03

**Authors:** David C. Alexander, Weilong Hao, Matthew W. Gilmour, Sandra Zittermann, Alicia Sarabia, Roberto G. Melano, Analyn Peralta, Marina Lombos, Keisha Warren, Yuri Amatnieks, Evangeline Virey, Jennifer H. Ma, Frances B. Jamieson, Donald E. Low, Vanessa G. Allen

**Affiliations:** Public Health Ontario, Toronto, Ontario, Canada (D.C. Alexander, W. Hao, S. Zittermann, R.G. Melano, A. Peralta, M. Lombos, K. Warren, J.H. Ma, F.B. Jamieson, D.E. Low, V.G. Allen);; University of Toronto, Toronto (D.C. Alexander, W. Hao, R.G. Melano, F.B. Jamieson, D.E. Low, V.G. Allen);; Mount Sinai Hospital, Toronto (W. Hao, K. Warren, D.E. Low);; Public Health Agency of Canada, Winnipeg, Manitoba, Canada (M.W. Gilmour);; Credit Valley Hospital, Mississauga, Ontario, Canada (A. Sarabia, Y. Amatnieks, E. Virey)

**Keywords:** *Escherichia coli*, Shiga toxin, genomics, bacteria, international travel, foodborne infections, travel-related infections, E. coli

## Abstract

We analyzed travel-associated clinical isolates of *Escherichia coli* O104:H4, including 1 from the 2011 German outbreak and 1 from a patient who returned from the Philippines in 2010, by genome sequencing and optical mapping. Despite extensive genomic similarity between these strains, key differences included the distribution of toxin and antimicrobial drug–resistance determinants.

In May 2011, officials in northern Germany reported a sudden surge in illness due to Shiga-toxigenic *Escherichia coli* (STEC). Symptoms of infection ranged from self-limiting episodes of diarrhea to life-threatening hemolytic-uremic syndrome (HUS). As of July 21, 2011, >4,075 persons in 16 countries had become ill. The outbreak was associated with an unprecedented rate of HUS (908 [22.2%] of 4,075 STEC-infected persons), and 50 persons died (*1*).

STEC are foodborne and waterborne pathogens. Human illness is most often associated with *E. coli* O157:H7, but non-O157 serogroups are also being recognized as key agents of STEC disease (*2*–*5*). The recent German outbreak was caused by *E. coli* O104:H4. Unlike *E. coli* O157:H7, which has a characteristic, sorbitol nonfermenting phenotype that is readily detected by routine laboratory testing, non-O157 *E. coli* strains are difficult to distinguish from the nonpathogenic *E. coli* strains commonly found in stool specimens, and frontline laboratories in Canada do not routinely screen for them.

This study describes 2 cases of *E. coli* O104:H4 infection that were imported to Canada. One case was caused by a 2011 isolate associated with the recent German outbreak. The second isolate was identified in 2010. Phenotypic and genotypic features of these 2 strains are described.

## The Study

On June 1, 2011, a 67-year-old Canadian man sought treatment at an Ontario hospital with a 3-day history of bloody diarrhea. He had returned from Germany on May 27. He had no signs of HUS, and *E. coli* O157:H7 was not detected by routine testing. Clinical specimens from this patient were referred to the Public Health Ontario Laboratories for testing. Shiga toxin was detected by enzyme immunoassay (Meridian Biosciences, Inc., Cincinnati, OH, USA), and real–time PCR confirmed that the strain, named ON-2011, was positive for the *stx2* gene and negative for the *eae* gene (*3*,*6*). Biochemical and serologic testing confirmed that the isolate was *E. coli* serogroup O104:H4. The patient recovered uneventfully (*4*).

Before the May 2011 outbreak in Germany, a single isolate of *E. coli* O104:H4 had been identified in Ontario. That isolate, ON-2010, was recovered in June 2010 from a 10-month-old boy who had returned from the Philippines 2 days earlier. He was brought to the hospital with a 1-day history of vomiting and nonbloody diarrhea. A sorbitol-nonfermenting colony was recovered from a stool specimen, but it did not react with *E. coli* O157 antiserum. The specimen was referred to the Canadian National Microbiology Laboratory, which confirmed *E. coli* O104:H4. Retrospective PCR-based testing showed that this isolate was negative for *stx* and *eae*. The infant made a full and uneventful recovery.

Etest (AB BIODISK, Solna, Sweden) susceptibility testing was performed by using a standard inoculum (0.5–McFarland standard); agar dilution indicated that ON-2010 was pansusceptible, whereas ON-2011 was resistant to amikacin, tetracycline, trimethoprim/sulfamethoxazole, and extended-spectrum β–lactams. PCR results for CTX–M–15 and TEM–1 were negative for ON-2010 and positive for ON-2011 (*7*). The gene for tellurite resistance was also absent in ON-2010, but present in ON-2011. Although both isolates were of multilocus sequence type 678, pulsed-field gel electrophoresis profiles were distinct (Figure, panel A) (*8*). Patterns for ON-2011 (ENXAI.0024/ENBNI.0022, PulseNet Canada designations) were identical with those reported for the current outbreak strain from Germany, whereas the ON-2010 profiles (ECXAI.2585/ECBNI.0922) were distinct (*9*).

**Figure:**
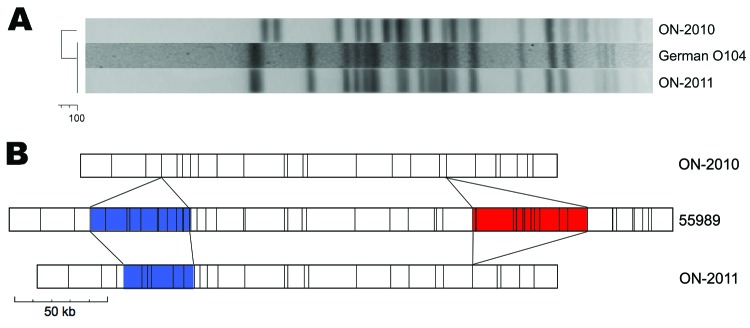
Comparison of *Escherichia coli* O104:H4 isolates from Ontario. A) The *Xba*I pulsed-field gel electrophoresis profile of ON-2010 is distinct from those of ON-2011 and the outbreak strain from Germany. B) Optical mapping (*Nco*I) patterns reveal genomic similarities and differences between ON-2010, ON-2011, and the O104:H4 strain 55989. Blue, heterogeneity in siderophore biosynthesis region; red, strain-specific insertion of TetR-containing prophage.

Circular, highresolution *Nco*I restriction maps of the ON-2010 (≈5.1 Mbp) and ON-2011 (≈5.25 Mbp) genomes were generated by optical mapping (Argus Optical Mapper, OpGen, Inc, Gaithersburg, MD, USA). These were compared with an in silico map of *E. coli* 55989, an O104:H4 strain that was isolated in Africa and sequenced in France (*10*). The extensive synteny observed and the absence of large-scale genomic rearrangements (e.g., inversions, translocations) suggest that ON-2010 and ON-2011 descended from an *E. coli* 55989–like ancestor (Figure, panel B).

The evolutionary relationship of the 2 isolates from Ontario was further assessed by whole-genome sequencing (Roche GS–FLX Titanium; Roche Diagnostics, Laval, QC, Canada). This confirmed the genomic similarity of ON-2010 to *E. coli* 55989 and uncovered a 72-kb plasmid, pON-2010. Virulence genes ON-2010 and ON-2011 were compared by Bielaszewska et al. (*11*) (Table) on the basis of virulence factor analysis of the 2011 outbreak strain of *E. coli* O104:H4 from Germany. The plasmid exhibits >99% identity with p55989 and encodes the aggregative adhesion fimbriae cluster that is a defining features of enteroaggregative *E. coli* (EAEC) (*12*). In contrast, ON-2011 contains 3 plasmids. Reference-based mapping of raw sequencing reads against publicly available genome and plasmid scaffolds (HPA, BGI) confirmed that ON-2011 is a German outbreak clone (*13*). Except for a small number of single nucleotide polymorphisms, the clone isolated in Canada is virtually identical to those from Germany and the United Kingdom.

**Table:** Virulence loci of ON-2011, ON-2010, and EAEC strain 55989*

Gene product	Function	Presence in ON-2011	Presence in ON-2010	Presence in EAEC 55989
STEC				
* stx_1_*	Shiga toxin 1	–	–-	–
* stx_2_*	Shiga toxin 2	+	–	–
EHEC-*hlyA*	EHEC hemolysin	–	–	–
*cdt*(I–V)	Cytolethal distending toxin	–	–	–
* subAB*	Subtilase cytotoxin	–	–	–
* espP*	Serine protease EspP	–	–	–
* eae*	Intimin	–	–	–
* iha*	Iha (IrgA homologue adhesin)	+	Partial (5′ end 21%)	+
* lpfA_O26_*	Structural subunit of LPF of STEC O26	+	+	+
* lpfA_O113_*	Structural subunit of LPF of STEC O113	+	+	+
* lpfA_O157–OI141_*	Structural subunit of LPF of STEC O157:H7 (encoded on O island 141)	–	–	–
* lpfA_O157–OI154_*	Structural subunit of LPF of STEC O157:H7 (encoded on O island 154)	–	–	–
* saa*	Saa (STEC autoagglutinating adhesin)	–	–	–
* sfpA*	Structural subunit of Sfp fimbriae	–	–	–
*ter* cluster	Tellurite resistance	+	–	–
* irp2*	Component of iron uptake system on HPI	+	+	+
* fyuA*	Component of iron uptake system on HPI	+	+	+
EAEC				
* aatA*	EAEC virulence plasmid (pAA)	+	+	+
* aggA*	Pilin subunit of aggregative adherence fimbriae I (AAF/I)	+	–	–
* agg3A*	Pilin subunit of AAF/III	–	+	+
* aggR*	Transcriptional regulator AggR	+	+	+
* aap*	Dispersin	+	+	+
* set1*	*Shigella* enterotoxin 1	+	+	+
* pic*	Pic (protein involved in intestinal colonization)	+	+	+
* astA*	EAEC heat-stable enterotoxin 1 (EAST1)	–	+	+
EPEC				
* bfpA*	Bundle-forming pili	–	–	–
ETEC				
* elt*	Heat-labile enterotoxin (LT)	–	–	–
* estla*	Heat-stable enterotoxin (STIa)	–	–	–
* estlb*	Heat-stable enterotoxin (STIb)	–	–	–
EIEC				
* ial*	Invasive plasmid (pInv)	–	–	–

*Derived from (*11*). EAEC, enteroaggregative *Escherichia coli;* STEC, Shiga- toxigenic *E. coli*; EHEC, enterohemorrhagic; LPF, long polar fimbriae; EPEC, enteropathogenic *E. coli;* ETEC, enterotoxigenic E. coli; EIEC, enterinvasive *E. coli.*

The O104:H4 outbreak strain from Germany exhibits key features of classic EAEC, but also contains numerous horizontally acquired virulence factors. An *stx2-*encoding prophage is responsible for the toxigenic properties of this strain, and multidrug-resistance loci are present, including a chromosomal tetR loci, and the plasmid-encoded CTX-M-15, TEM-1, and tellurite resistance–encoding loci (*ter*) (*14*). In contrast, the ON-2010 strain does not encode Shiga toxin, and no resistance genes have been identified. Even the tetracycline-resistance genes, independently acquired by both ON-2011 and strain 55989, are absent (Figure, panel B). However, the plasmid-encoding aggregative adhesion fimbriae cluster is present. Preliminary comparison of the 3 genomes has also shown some small-scale rearrangement events. One of these rearrangements eliminates siderophore biosynthesis genes from ON-2010 and may compromise bacterial iron acquisition. However, ON-2010 does have a strain-specific insertion that contains the hydroxyphenylacetate (*hpa*) operon, which may provide a nutritional advantage by enabling the organism to catabolize the phenolic and aromatic compounds abundant in the gut. This insertion is absent from the other O104:H4 genomes (*15*).

## Conclusions

Since 2010, 2 cases of travel-associated *E. coli* O104:H4 infection have been identified in Ontario, Canada. Our analysis of these isolates could inform genomic studies on the emergence and evolution of the O104 clone first observed in Europe. The relationship of these strains was assessed by a combination of traditional, molecular, and genomic approaches. Optical mapping and sequencing indicate that ON-2010 and ON-2011 exhibit extensive synteny and are derived from a common EAEC ancestor. However, a series of horizontal gene transfer events has contributed to genotypic and phenotypic divergence. The outbreak clone from Germany, ON-2011, is an antimicrobial drug–resistant STEC isolate, whereas ON-2010 is pansusceptible and nontoxigenic.

These differences are clinically important but are not detected by diagnostic strategies that use serotype as a proxy for pathogenic capacity. Given the potential for severe clinical sequelae of STEC infections, clinical testing should use improved and affordable methods for identification of Shiga toxin that can be easily integrated into routine clinical microbiology laboratories (*4*,*9*). Future capacity to detect additional virulence factors, such as aggregative adhesion fimbriae, would enable expanded diagnostic capacity to detect pathogenic *E. coli* subtypes that cause human disease.
